# Features of Epstein–Barr Virus and Cytomegalovirus Reactivation in Acute Leukemia Patients After Haplo-HCT With Myeloablative ATG-Containing Conditioning Regimen

**DOI:** 10.3389/fcimb.2022.865170

**Published:** 2022-05-16

**Authors:** Yuhua Ru, Jinjin Zhu, Tiemei Song, Yiyang Ding, Ziling Zhu, Yi Fan, Yang Xu, Aining Sun, Huiying Qiu, Zhengming Jin, Xiaowen Tang, Yue Han, Chengcheng Fu, Suning Chen, Xiao Ma, Feng Chen, Jia Chen, Depei Wu

**Affiliations:** ^1^ National Clinical Research Center for Hematologic Diseases, Jiangsu Institute of Hematology, The First Affiliated Hospital of Soochow University, Suzhou, China; ^2^ Institute of Blood and Marrow Transplantation, Collaborative Innovation Center of Hematology, Soochow University, Suzhou, China; ^3^ Key Laboratory of Stem Cells and Biomedical Materials of Jiangsu Province and Chinese Ministry of Science and Technology, Suzhou, China

**Keywords:** Epstein–Barr virus (EBV), cytomegalovirus (CMV), acute leukemia, haplo-HCT, outcome

## Abstract

**Background:**

Haploidentical donor hematopoietic cell transplantation (haplo-HCT) has become a preferred option for patients without HLA-matched donors, but it increases the risk of viral reactivations. Epstein–Barr virus (EBV) and cytomegalovirus (CMV) are common viruses post-HCT, but limited data have been reported in the setting of haplo-HCT.

**Methods:**

We conducted a retrospective study enrolling acute leukemia patients who received haplo-HCT with myeloablative conditioning regimen employing ATG in our center from July 2014 to July 2017. All the patients enrolled were EBV-IgM and EBV-DNA negative but EBV-IgG positive, and so were their donors. The same went for CMV as well.

**Results:**

In total, 602 patients were recruited consisting of 331 with acute myeloid leukemia (AML) and 271 with acute lymphoblastic leukemia (ALL). One-year cumulative incidences of EBV (22.9% ± 2.4% vs. 27.4% ± 2.8%, *P* = 0.169) and CMV (24.7% ± 2.4% vs. 29.4% ± 2.8%, *P* = 0.190) reactivation were comparable between AML and ALL. EBV and CMV were independent risk factors for each other. In the AML group, male recipients [HR = 1.275, 95% CI (1.001–1.624), *P* = 0.049] and acute graft-versus-host disease [HR = 1.592, 95% CI (1.001–2.533), *P* = 0.049] were independent risk factors for EBV reactivation and CMV reactivation, respectively. CMV rather than EBV reactivation was related to a trend of worsened treatment-related mortality (TRM) (15.6% ± 0.1% vs. 10.2% ± 0.0%, *P* = 0.067) and progression-free survival (PFS) (60.6% ± 4.1% vs. 70.3% ± 2.3%, *P* = 0.073), while significant impacts were revealed only in the subgroup analysis. CMV reactivation resulted in a remarkable inferior 2-year overall survival (OS) (64.2% ± 5.7% vs. 77.6% ± 3.2%, *P* = 0.038) and PFS (55.0% ± 5.9% vs. 71.9% ± 3.4%, *P* = 0.042) in ALL patients. On the other hand, in the EBV+/CMV− subgroup, relapse was lower in ALL patients (8.2% ± 0.2% vs. 32.4% ± 0.8%, *P* = 0.010) compared with AML patients, which led to a superior 2-year OS (82.0% ± 6.2% vs. 60.3% ± 8.8%, *P* = 0.016) and PFS (74.5% ± 7.0% vs. 57.5% ± 8.4%, *P* = 0.036).

**Conclusion:**

We concluded that EBV and CMV reactivations were frequent in acute leukemia patients after haplo-HCT, with possibly distinctive risk factors from HLA-matched HCT. There could be a potential interaction between EBV and CMV, but impacts on transplant outcomes remained complex.

## Introduction

Viral reactivation remains a major concern for recipients of allogeneic hematopoietic cell transplantation (allo-HCT) (Gratwohl et al., 2005; Styczynski et al., 2009; Locatelli et al., 2013; Gilis et al., 2014; Jain et al., 2014). Epstein–Barr virus (EBV) and cytomegalovirus (CMV) reactivations after allo-HCT are frequent complications that can lead to the deadliest virus-related diseases (Sundin et al., 2006; Ru et al., 2018; Giménez et al., 2019), so they are regularly monitored in most of the transplant centers. Considerable literature described the prevalence and the prognostic impact of both EBV and CMV reactivations. However, the reported incidence widely varied, and the impact on the prognosis of either CMV or EBV remained controversial (Behrendt et al., 2009; Elmaagacli et al., 2011; Peric et al., 2011; Hoegh-Petersen et al., 2012; Green et al., 2013; Auger et al., 2014; Manjappa et al., 2014; Uhlin et al., 2014; Jang et al., 2015; Li et al., 2016; Teira et al., 2016; Ru et al., 2020). Moreover, there could be a potential interaction between EBV and CMV reactivation, but few data regarding the co-reactivation were reported particularly in alternative donor HCT.

Human leukocyte antigen (HLA)-haploidentical HCT (haplo-HCT) is a valuable treatment option for patients who lack a suitable HLA-matched donor (Kanda et al., 2010; Passweg et al., 2015; Kanakry et al., 2016; Passweg et al., 2017; Xu et al., 2017; Al Malki et al., 2018). The reliable accessibility to the donors has resulted in a booming number of haplo-HCT worldwide, despite an increased risk for viral reactivation (Raj et al., 2016) even with the improved virus management in the modern era. On account of the ambiguous results of limited studies focusing on the co-reactivation of EBV and CMV after haplo-HCT, we conducted a retrospective analysis to preliminarily describe the features of co-reactivation of EBV and CMV in a group of acute leukemia patients undergoing haplo-HCT.

## Patients and Methods

### Patients

This was a retrospective study based on the data derived from the transplant database of our center, which was established according to the European Society for Blood and Marrow Transplantation (EBMT) registry. The patient inclusion criteria were as follows: i) patients who underwent haplo-HCT in our center from July 2014 to July 2017, ii) patients who were diagnosed with acute leukemia (except acute promyelocytic leukemia), and iii) patients who received regular EBV and CMV management according to institutional protocol. The study protocol was approved by the ethics committee of our center and followed the tenets of the Declaration of Helsinki.

### Donor and Graft Selection and Transplant Protocols

For haplo-HCT, a young male donor was the optimal choice, whereas a maternal donor (mother donor, MD) or a collateral relative donor (CRD, e.g., uncle, aunt, nephew, niece, and cousin) served as alternative options (Wang et al., 2014; Zhang et al., 2014; Xu et al., 2018). The donors were recommended to contribute graft of bone marrow, complemented with peripheral blood stem cells (PBSCs) if the CD34^+^ cell dose failed to achieve the target dose of 2 × 10^6^/kg of recipient body weight. All of the enrolled patients received myeloablative conditioning with a modified Bu/Cy regimen (Chen et al., 2018).

### Management of Graft-Versus-Host Disease

Prophylaxis of graft-versus-host disease (GVHD) was composed of cyclosporin A (CsA) and short-term methotrexate (MTX), mycophenolate mofetil (MMF), and antithymocyte globulin (ATG) (Genzyme, MA, USA). The diagnosis of acute and chronic GVHD was established according to reference literature (Przepiorka et al., 1995; Jagasia et al., 2015). Methylprednisolone at a dose of 1~2 mg/kg/day was administered as the first-line treatment for overt acute GVHD occurrence. The second-line drugs included tacrolimus, anti-CD25 monoclonal antibody, MMF, ATG, etc. The first-line treatment of overt chronic GVHD was steroids and/or CsA.

### Management of EBV and CMV Reactivation

qPCR was applied to monitor EBV-DNA and CMV-DNA copies with the sample of whole peripheral blood weekly from hematopoietic recovery to day +90 post-HCT in all the patients and once every 2 weeks from +90 days until +180 days. Additional detection was performed if symptoms of suspected virus infection were present. All the patients enrolled in this study were EBV-IgM and EBV-DNA negative but EBV-IgG positive due to the distinctiveness of the Chinese population. All of the donors of the enrolled recipients were the same in serostatus. The same went for CMV as well. Therefore, we did not analyze the impact of pretransplant EBV and CMV serostatus of the patients and their donors. Ganciclovir at a dose of 10 mg/kg/day was used from day 9 to day 2 before HCT to prevent virus infection and then replaced by acyclovir to avoid marrow toxicity. Intravenous immunoglobulin (IVIG) was also recommended weekly as prophylaxis with a dose of 0.4 g/kg of recipient body weight in the first 3 months. The treatment for EBV or CMV reactivated recipients included tapering of immunosuppressive agents, ganciclovir, and foscarnet sodium. Furthermore, preemptive rituximab was given if EBV-DNA reached 10^5^ copies/ml or 10^4^ for consecutive 2 weeks, while IVIG was daily prescribed if EBV or CMV diseases developed.

### Definition

In our center, reactivation of both EBV and CMV was defined as more than 10^2^ copies/ml EBV-DNA or CMV-DNA in the whole peripheral blood by qPCR in two consecutive tests. The survival time was calculated from the day of transplantation. Overall survival (OS) was calculated until the date of death or last follow-up or study end. Progression-free survival (PFS) was calculated until death, disease progression, or last follow-up, whichever occurred first. Deaths irrelative with acute leukemia were recorded as treatment-related mortality (TRM). Cumulative incidence of relapse (CIR) was calculated until the relapse of leukemia.

### Statistics

For comparisons of baseline characteristics, continuous variables were compared by the independent Kruskal–Wallis test, and category variables were compared by the chi-square test. In risk analyses, all predictors with a *P*-value below 0.10 in the univariate analysis were included in the multivariate analysis. Events post-HCT such as viral reactivation and GVHD were treated as time-dependent variables. OS and PFS were calculated using the Kaplan–Meier method and compared with the log-rank test. CIR was calculated by a competing risk model with TRM as a competing risk factor. All *P*-values are two-sided and defined as statistically significant if *P*-value is less than 0.05. Statistical analyses were performed using SPSS 19.0 software (SPSS, Chicago, IL, USA) and R 3.6.1 software package (The R Foundation for Statistical Computing, Vienna, Austria).

## Results

### Patients’ Characteristics

A total of 602 patients were enrolled in this study according to the inclusion criteria, consisting of 331 cases with acute myeloid leukemia (AML) and 271 cases with acute lymphoblastic leukemia (ALL). The baseline characteristics are summarized in **Table 1**. The enrolled patients consisted of 356 men and 246 women, and the median age at the time of haplo-HCT was 27 (range, 1–65). There were only significant differences between AML and ALL patients in terms of age (*P* < 0.001), period (*P* < 0.001), and disease status pre-HCT (*P* = 0.001). The enrolled AML patients were older than ALL patients and there were more AML patients with an advanced disease.

**Table 1:** Characteristics of patients between the AML group and the ALL group.

	AML	ALL	*P*
**Sex**			0.954
Female	144	102	
Male	187	169	
**Age (years)**			<0.001
<25	110	152	
≥25	221	119	
**Period**			<0.001
Adult	264	168	
Children	67	103	
**Disease status before HCT**			0.001
CR1 or CR2	267	246	
CR3 or beyond	64	25	
**Acute GVHD**			0.546
Grades 0–1	249	198	
Grades 2–4	82	73	
**Chronic GVHD**			0.471
Absent	223	175	
Present	108	96	
**EBV reactivation**			0.225
Positive	79	77	
Negative	252	196	
**CMV reactivation**			0.212
Positive	85	82	
Negative	246	189	

AML, acute myeloid leukemia; ALL, acute lymphoblastic leukemia; CR, complete remission; GVHD, graft-versus-host disease; EBV, Epstein–Barr virus; CMV, cytomegalovirus.

### Prevalence of Viral Reactivation

In the whole cohort, EBV reactivation occurred in 156 cases with the median time of 59 (range, 19–703) days post-HCT, while CMV reactivation occurred in 167 cases with the median time of 55 (range, 18–1,146) days post-HCT, respectively. The 1-year cumulative incidence was 24.9% ± 1.8% for EBV reactivation and 26.4% ± 1.8% for CMV reactivation, respectively.

In the AML group, EBV reactivation occurred in 79 cases with the median time of 56 (range, 19–654) days post-HCT, while CMV reactivation occurred in 85 cases with the median time of 57 (range, 22–900) days post-HCT, respectively. In the ALL group, EBV reactivation occurred in 77 cases with the median time of 59 (range, 24–703) days post-HCT, while CMV reactivation occurred in 82 cases with the median time of 53 (range, 18–441) days post-HCT, respectively. There were no statistically significant differences with respect to the 1-year cumulative incidence of EBV reactivation (22.9% ± 2.4% vs. 27.4% ± 2.8%, *P* = 0.169) and CMV reactivation (24.7% ± 2.4% vs. 29.4% ± 2.8%, *P* = 0.190) between the two groups.

### Risk Factors for Viral Reactivation

In the univariate analysis, male patients [HR = 1.824, 95% CI (1.129–2.946), *P* = 0.014] and CMV reactivation [HR = 3.751, 95% CI (2.369–5.941), *P* < 0.001] had a significant association with EBV reactivation in the AML group, while acute GVHD had a statistical trend [HR = 1.602, 95% CI (0.985–2.605), *P* = 0.057] (**Table S1**). Three factors that had a significant association with CMV reactivation were identified: male patients [HR = 1.694, 95% CI (1.073–2.675), *P* = 0.024], acute GVHD [HR = 1.780, 95% CI (1.122–2.825), *P* = 0.014], and EBV reactivation [HR = 3.948, 95% CI (2.531–6.156), *P* < 0.001] (**Table S2**). Chronic GVHD [HR = 3.028, 95% CI (1.020–8.988), *P* = 0.046] and CMV reactivation [HR = 2.069, 95% CI (1.289–3.319), *P* = 0.003] were associated with EBV reactivation after HCT in the ALL group (**Table S3**), EBV reactivation [HR = 1.979, 95% CI (1.205–3.250), *P* = 0.007] was associated with CMV reactivation, while chronic GVHD [HR = 2.939, 95% CI (0.863–10.007), *P* = 0.085] had a marginal significance (**Table S4**).

In the multivariate analysis, it was intriguing that EBV reactivation and CMV reactivation were independent risk factors for each other in both AML and ALL patients. CMV reactivation [HR = 3.421, 95% CI (2.136–5.479), *P* < 0.001] and male patients [HR = 1.275, 95% CI (1.001–1.624), *P* = 0.049] independently increased the risk of EBV reactivation in the AML group, while EBV reactivation [HR = 3.606, 95% CI (2.300–5.654), *P* < 0.001] and acute GVHD [HR = 1.592, 95% CI (1.001–2.533), *P* = 0.049] were independent risk factors for CMV reactivation. In ALL patients, CMV reactivation [HR = 2.003, 95% CI (1.246–3.220), *P* = 0.004] was an independent risk factor for EBV reactivation and vice versa [HR = 1.975, 95% CI (1.202–3.244), *P* = 0.007]. No additional independent risk factors for EBV or CMV reactivation were found in ALL patients (**Table 2**).

**Table 2:** Multivariate Cox regression model for EBV reactivation and CMV reactivation.

	AML			ALL		
	Factor	HR (95% CI)	*P*	Factor	HR (95% CI)	*P*	
**EBV**	CMV+	3.421 (2.136–5.479)	<0.001	CMV+	2.003 (1.246–3.220)	0.004	
	Male	1.275 (1.001–1.624)	0.049	Chronic GVHD	2.725 (0.907–8.187)	0.074	
	Acute GVHD	1.272 (0.773–2.092)	0.343			
**CMV**	EBV+	3.606 (2.300–5.654)	<0.001	EBV+	1.975 (1.202–3.244)	0.007	
	Acute GVHD	1.592 (1.001–2.533)	0.049	Chronic GVHD	2.919 (0.857–0.939)	0.074	
	Male	1.221 (0.970–1.537)	0.089			

AML, acute myelocytic leukemia; ALL, acute lymphoblastic leukemia; EBV, Epstein–Barr virus; CMV, cytomegalovirus; EBV+, EBV reactivation; CMV+, CMV reactivation; GVHD, graft-versus-host disease; HR, hazard ratio.

### Impact of EBV and CMV Reactivation on Transplant Outcomes

The median follow-up of all the enrolled acute leukemia patients was 23 (range, 0–61) months. Neither EBV nor CMV reactivation had a significant impact on outcomes in the whole cohort, despite trends toward deterioration of 2-year TRM (15.6% ± 0.1% vs. 10.2% ± 0.0%, *P* = 0.067) and PFS (60.6% ± 4.1% vs. 70.3% ± 2.3%, *P* = 0.073) for those developing CMV reactivation post-HCT (**Table 3**). For AML patients, it seemed that EBV reactivation was related to a decreased 2-year CIR (0.7% ± 0.3% vs. 12.3% ± 0.1%, *P* = 0.088), while CMV reactivation led to a slightly higher 2-year TRM (14.6% ± 0.2% vs. 9.1% ± 0.0%, *P* = 0.099). Nevertheless, OS and PFS were comparable for AML patients regardless of the virus status (**Table 3**). For ALL patients, EBV had little impact on transplant outcomes, and CMV reactivation post-HCT insignificantly increased the 2-year TRM (18.2% ± 0.2% vs. 11.1% ± 0.1%, *P* = 0.195) and CIR (19.6% ± 0.2% vs. 16.1% ± 0.1%, *P* = 0.313). Of note, ALL patients who developed CMV reactivation had a remarkable inferior 2-year OS (64.2% ± 5.7% vs. 77.6% ± 3.2%, *P* = 0.038) and PFS (55.0% ± 5.9% vs. 71.9% ± 3.4%, *P* = 0.042) (**Table 3**).

**Table 3:** Comparisons of transplant outcomes of patients with or without viral reactivations post-HCT.

		2-Year OS	2-Year PFS	2-Year TRM	2-Year CIR
**The whole cohort**					
**EBV reactivation**	**Positive**	69.8% ± 3.9%	63.7% ± 4.0%	11.0% ± 0.1%	18.5% ± 0.1%
	**Negative**	76.5% ± 2.1%	69.1% ± 2.3%	11.7% ± 0.0%	13.2% ± 0.0%
	***P***	0.273	0.342	0.617	0.193
**CMV reactivation**	**Positive**	70.5% ± 3.8%	60.6% ± 4.1%	15.6% ± 0.1%	18.6% ± 0.1%
	**Negative**	76.2% ± 2.1%	70.3% ± 2.3%	10.2% ± 0.0%	14.1% ± 0.0%
	***P***	0.253	0.073	0.067	0.375
**AML patients**					
**EBV reactivation**	**Positive**	67.9% ± 5.6%	63.1% ± 5.6%	11.3% ± 0.2%	20.7% ± 0.3%
	**Negative**	77.6% ± 2.7%	70.3% ± 3.0%	10.2% ± 0.0%	12.3% ± 0.1%
	***P***	0.137	0.156	0.358	0.088
**CMV reactivation**	**Positive**	75.1% ± 5.0%	66.3% ± 5.4%	14.6% ± 0.2%	16.5% ± 0.2%
	**Negative**	75.1% ± 2.9%	69.1% ± 3.0%	9.1% ± 0.0%	13.6% ± 0.1%
	***P***	0.777	0.616	0.099	0.570
**ALL patients**					
**EBV reactivation**	**Positive**	71.7% ± 5.4%	64.5% ± 5.8%	15.6% ± 0.2%	15.1% ± 0.2%
	**Negative**	75.0% ± 3.3%	67.7% ± 3.5%	12.1% ± 0.1%	13.9% ± 0.1%
	***P***	0.980	0.855	0.764	0.974
**CMV reactivation**	**Positive**	64.2% ± 5.7%	55.0% ± 5.9%	18.2% ± 0.2%	19.6% ± 0.2%
	**Negative**	77.6% ± 3.2%	71.9% ± 3.4%	11.1% ± 0.1%	16.1% ± 0.1%
	***P***	0.038	0.042	0.195	0.313

HCT, hematopoietic cell transplantation; EBV, Epstein–Barr virus; CMV, cytomegalovirus; OS, overall survival; PFS, progression-free survival; TRM, treatment-related mortality; CIR, cumulative incidence of relapse; AML, acute myeloid leukemia; ALL, acute lymphoblastic leukemia.

### Subgroup Analyses Based on Viral Reactivation

**Figure 1:**
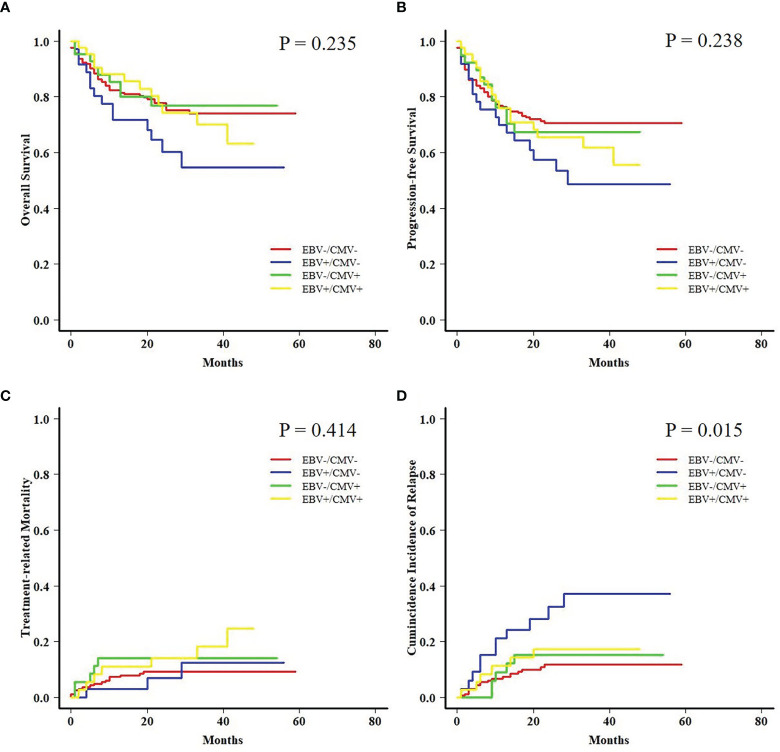
Transplant outcomes among subgroups based on viral reactivation in acute myeloid leukemia (AML) patients. **(A)** Overall survival (OS) among subgroups based on viral reactivation in AML patients. **(B)** Progression-free survival (PFS) among subgroups based on viral reactivation in AML patients. **(C)** Treatment-related mortality (TRM) among subgroups based on viral reactivation in AML patients. **(D)** Cumulative incidence of relapse (CIR) among subgroups based on viral reactivation in AML patients.

**Figure 2:**
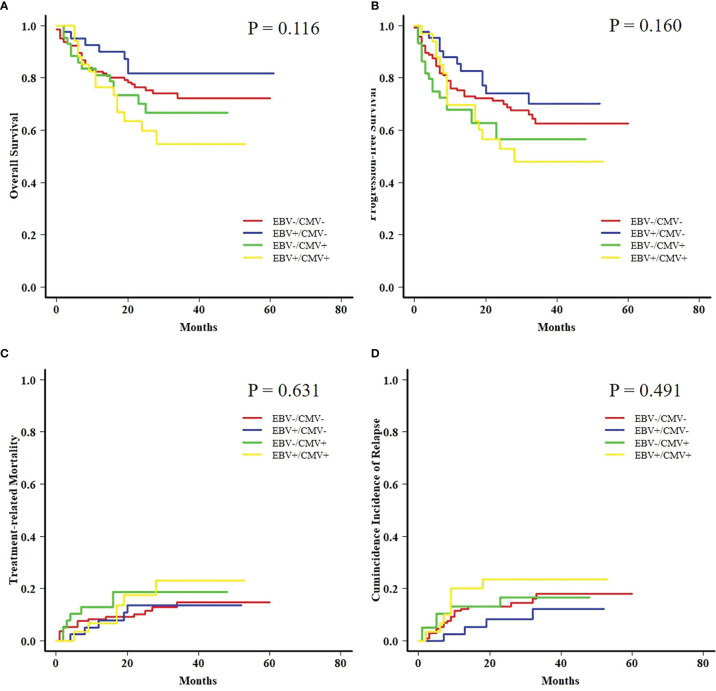
Transplant outcomes among subgroups based on viral reactivation in acute lymphoblastic leukemia (ALL) patients. **(A)** OS among subgroups based on viral reactivation in ALL patients. **(B)** PFS among subgroups based on viral reactivation in ALL patients. **(C)** TRM among subgroups based on viral reactivation in ALL patients. **(D)** CIR among subgroups based on viral reactivation in ALL patients.

Both AML and ALL patients were respectively divided into four subgroups according to the status of EBV and CMV co-reactivation: EBV−/CMV−, EBV+/CMV−, EBV−/CMV+, and EBV+/CMV+. For AML patients, OS, PFS, and TRM had no difference among the four subgroups, but patients in the EBV+/CMV− subgroup had a significantly increased CIR (*P* = 0.015) than the other three subgroups (**Figure 1**). Interestingly, ALL patients in the EBV+/CMV– subgroup seemed to have a better OS (*P* = 0.116), PFS (*P* = 0.160), and CIR (*P* = 0.491) than those in the other subgroups, although without statistical significance (**Figure 2** and **Table 4**).

**Table 4:** Transplant outcomes of patients among subgroups based on viral reactivation.

	EBV−/CMV−	EBV+/CMV−	EBV−/CMV+	EBV+/CMV+	*P*
**AML**			**AML**
***n***	209	37	43	42	Total: 331
**OS**	77.8% ± 3.0%	60.3% ± 8.8%	76.8% ± 6.9%	74.3% ± 7.1%	0.235
**PFS**	70.7% ± 3.2%	60.0% ± 8.4%	67.4% ± 7.8%	65.7% ± 7.5%	0.238
**TRM**	9.3% ± 0.1%	6.9% ± 0.2%	14.1% ± 0.4%	14.1% ± 0.4%	0.414
**CIR**	11.6% ± 0.1%	32.4% ± 0.8%	15.3% ± 0.4%	17.2% ± 0.4%	0.015
**ALL**			**ALL**
***n***	148	41	46	36	Total: 271
**OS**	76.5% ± 3.7%	81.7% ± 6.3%	70.2% ± 7.3%	59.8% ± 8.8%	0.116
**PFS**	71.2% ± 3.9%	74.2% ± 7.1%	56.5% ± 7.9%	52.9% ± 9.0%	0.160
**TRM**	10.2% ± 0.1%	13.6% ± 0.3%	18.6% ± 0.4%	17.6% ± 0.5%	0.631
**CIR**	13.1% ± 0.1%	8.2% ± 0.2%	16.7% ± 0.4%	23.5% ± 0.6%	0.491

EBV, Epstein–Barr virus; CMV, cytomegalovirus; AML, acute myeloid leukemia; ALL, acute lymphoblastic leukemia; OS, overall survival; PFS, progression-free survival; TRM, treatment-related mortality; CIR, cumulative incidence of relapse.

**Figure 3:**
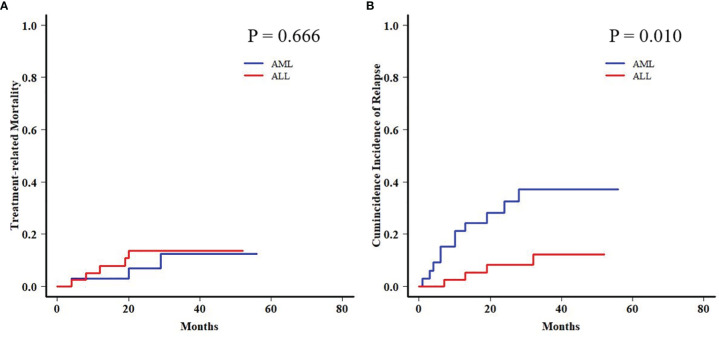
Transplant outcomes between AML and ALL patients in the Epstein–Barr virus (EBV)+/cytomegalovirus (CMV)− subgroup. **(A)** TRM between AML and ALL patients in the EBV+/CMV− subgroup. **(B)** CIR between AML and ALL patients in the EBV+/CMV− subgroup.

**Figure 4:**
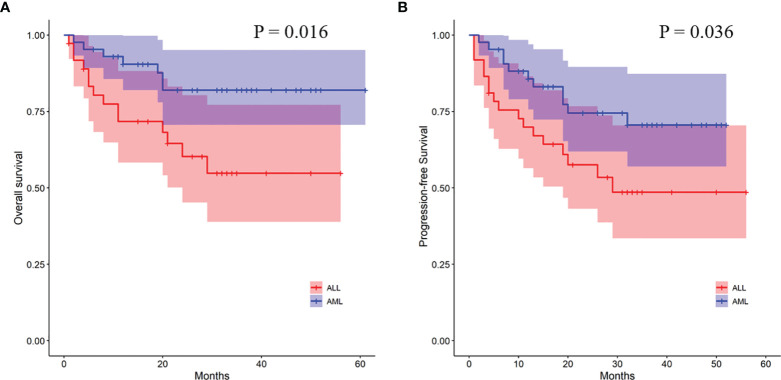
Transplant outcomes between AML and ALL patients in the EBV+/CMV− subgroup. **(A)** OS between AML and ALL patients in the EBV+/CMV− subgroup. **(B)** PFS between AML and ALL patients in the EBV+/CMV− subgroup.

In order to further analyze the patients with EBV+/CMV− status, we compared the transplant outcomes of patients with AML or ALL both in this subgroup and in the whole cohort. As expected, OS, PFS, and TRM were similar between AML and ALL patients in the whole cohort as well as a potentially increased 2-year CIR in the latter ones (12.1% ± 0.0% vs. 15.9% ± 0.1%, *P* = 0.081) (**Table S5** and **Figure S1**). In contrast to the results of the whole cohort, ALL patients had a comparable 2-year TRM but a markedly reduced CIR (8.2% ± 0.2% vs. 32.4% ± 0.8%, *P* = 0.010) (**Figure 3**) than AML patients in the EBV+/CMV− subgroup, which accordingly brought a superior 2-year OS (82.0% ± 6.2% vs. 60.3% ± 8.8%, *P* = 0.016) and PFS (74.5% ± 7.0% vs. 57.5% ± 8.4%, *P* = 0.036) (**Table S5** and **Figure 4**). In the univariate Fine and Gray model for CIR in this subgroup, AML was identified as the only risk factor for relapse (**Table S6**).

**Figure 5:**
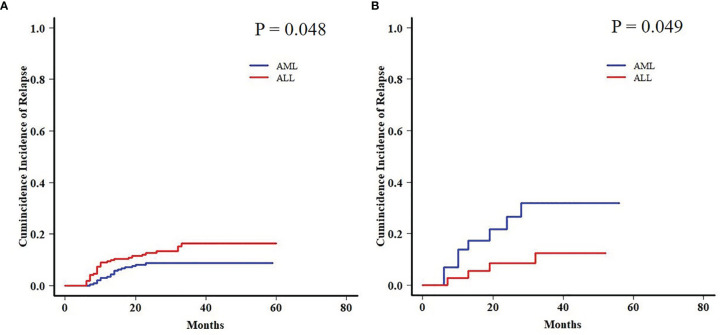
Late effect on CIR between AML and ALL patients. **(A)** Late effect on CIR between AML and ALL patients in the whole cohort. **(B)** Late effect on CIR between AML and ALL patients in the EBV+/CMV− subgroup.

Moreover, a late effect on CIR was shown in patients who survived for more than 6 months post-HCT. AML patients in the EBV+/CMV− subgroup still had an increased 2-year CIR when compared with ALL patients (31.8% ± 1.0% vs. 12.4% ± 0.4%, *P* = 0.049), contrary to the results in the whole cohort again (8.7% ± 0.0% vs. 16.3% ± 0.1%, *P* = 0.048) (**Figure 5**).

## Discussion

Allo-HCT is a potentially curative treatment modality for most patients with malignant and non-malignant hematological disorders (Copelan, 2006), and acute leukemia is the main indication. However, all patients have no available HLA-matched sibling or unrelated donors. Given the feasible accessibility to the donor as well as reliable engraftment and graft-versus-leukemia (GVL) effect, a haploidentical donor has become the optimal alternative donor in recent years (Kanda et al., 2010; Passweg et al., 2015; Kanakry et al., 2016; Passweg et al., 2017; Xu et al., 2017; Al Malki et al., 2018). The “Beijing protocol” is currently the most popular haplo-HCT conditioning regimen in China employing ATG for T-cell depletion (TCD) *in vivo* (Wang et al., 2021), while the use of both the ATG and haploidentical donor will exacerbate the risk of viral reactivation after transplantation (Walker et al., 2007; Landgren et al., 2009; Yoon et al., 2009; Uhlin et al., 2014; Qayed et al., 2015; Raj et al., 2016; Ru et al., 2020). Although management strategies for viral reactivation in immunocompromised patients are well established, EBV and CMV reactivations and subsequent tissue-invasive diseases remain challenging for post-HCT patients. The considerable mortality of EBV and CMV diseases has been little ameliorated in recent years (Camargo and Komanduri, 2017; Mehta Steinke et al., 2021), so it is necessary to learn the prevalence of viral reactivation which may benefit the improvement of the management strategy. Thereafter, we conducted a retrospective study to analyze the features of both EBV and CMV reactivations after haplo-HCT in patients with acute leukemia.

The population who received haploidentical grafts themselves was a high-risk group for viral reactivation, but there were very little data to clarify the viral reactivation, especially co-reactivation in this group. Therefore, we analyzed the characteristics of viral reactivation and the impact of co-reactivation in this high-risk population to compensate for the lack of data in this segment. The increased risk of viral reactivation after haplo-HCT is supposed to arise from the inevitable TCD *in vivo* and *in vitro*. The immunosuppression effect of ATG can eliminate T cells to inhibit the cellular immune function and prolong the immunosuppression period post-HCT, facilitating the viral reactivation (Kanda et al., 2010). The reported incidence of EBV reactivation after HCT ranged from 0.1% to 63% (Ocheni et al., 2008; Styczynski et al., 2016; Gao et al., 2019; Ru et al., 2020; Zhou et al., 2020a; Zhou et al., 2020b), and few data regarding CMV reactivation had been displayed. The incidence of viral reactivation in our study was at a relatively lower level compared with haplo-HCT studies, probably owing to the rigorous management in our center, but still higher than that after HLA-matched donor HCT as reported in large-scale studies (Ocheni et al., 2008; Ru et al., 2020). Furthermore, we previously found that the haploidentical donor had an additional risk for EBV reactivation independent of the use of ATG (Ru et al., 2020), which might explain the delayed median onset time of viral reactivation after haplo-HCT. The distinctive pattern of immunoreconstitution after haplo-HCT may lead to a prolonged susceptibility to viral reactivation. Thus, it is suggested to regularly monitor the virus status at least 6 months post-HCT according to our results.

Previous studies reported a series of impactors for viral reactivation in recipients after allo-HCT, including haploidentical donor, ATG use, age, and GVHD (Landgren et al., 2009; Uhlin et al., 2014; Ru et al., 2020). Although patients receiving haplo-HCT generally bear a higher risk of viral reactivation, it is still meaningful to further define the risk category in this group of patients. In our study, risk analyses were performed in AML and ALL patients, respectively, because of the apparent differences in biology and therapies. It was noted that EBV and CMV reactivation had a mutual impact across the type of acute leukemia, which increased the risk of each other. Co-reactivation accounted for 31.8% (78/245) among the patients with either viral reactivation in our study, and the association between EBV and CMV reactivation was also observed in other studies (Zallio et al., 2013; Papadopoulou et al., 2014; Gao et al., 2019). It was mainly attributed to deeper immunosuppression caused by delayed immunoreconstitution, infections, or anti-GVHD agents, favoring the reactivation of various viruses. Moreover, interactions between EBV and CMV reactivation in immunocompromised patients were also explored (Zallio et al., 2013). Another independent risk factor related to immunosuppression was GVHD (Matthes-Martin et al., 2003; Asano-Mori et al., 2005; Zallio et al., 2013), which was consistent with the finding of Yoon et al. (2009). Immunosuppressive agents against GVHD might also increase the risk of viral reactivation, but the impact of acute or chronic GVHD on either EBV or CMV reactivation varied in AML and ALL patients. Furthermore, it seemed that male patients with AML had a higher incidence of EBV reactivation (*P* = 0.049) and a potentially higher incidence of CMV reactivation (*P* = 0.089). Compared with previous studies in allo-HCT, fewer risk factors for viral reactivation were found in our study probably due to the high-risk nature of haplo-HCT. New biomarkers for viral reactivation are warranted to be explored, and our findings need further validation in the future.

The impacts of EBV and CMV reactivation on transplant outcomes were controversial. Our results suggested that EBV reactivation might elevate the risk of relapse in AML patients (*P* = 0.088), while CMV reactivation could worsen the OS (*P* = 0.038) and PFS (*P* = 0.042) in ALL patients. CMV reactivation was considered to be a negative impactor for TRM and survival, which was in accordance with Mariotti et al. (2022). However, they concluded that patient CMV serostatus is the main predictor of CMV reactivation. Although all of the donors of the enrolled recipients were the same in serostatus of CMV and EBV, it still should be considered when evaluating strategies for preventing CMV reactivation in further studies. There were also a handful of studies exhibiting the association of CMV reactivation with a mitigated risk of relapse after transplantation in AML patients (Behrendt et al., 2009; Elmaagacli et al., 2011; Green et al., 2013; Manjappa et al., 2014; Jang et al., 2015). On the contrary, a recently published study from the Center for International Blood and Marrow Transplant Research (CIBMTR) retrospectively analyzed 11,153 patients, including 5,310 AML patients, and found that CMV reactivation had no preventive effect on hematologic disease relapse irrespective of diagnosis (Teira et al., 2016).

With concerns about the ambiguous impact of co-reactivation, the analyses were further performed among fractionized subgroups. In the AML group, it was unexpected that patients with EBV+/CMV− had a significantly higher CIR than the other three subgroups. The intersubgroup comparison showed that for those with EBV+/CMV−, AML patients had drastically increased CIR and therefore a poorer OS and PFS when compared with ALL patients. This finding was further validated by a Fine and Gray model for CIR in this subgroup that identified AML as the only risk factor (**Table S6**), as well as a contrast with inverse results in the whole cohort (**Table S5**). A late effect of viral reactivation on transplant outcomes was reported by the research conducted by the Japanese Society for Hematopoietic Cell Transplantation (Takenaka et al., 2015), and this was also observed in our previous studies (Ru et al., 2020; Ding et al., 2021). In this study, for patients surviving more than 6 months post-HCT, the CIR was higher for those with ALL in the whole cohort (*P* = 0.048) but lower in the EBV+/CMV− subgroup (*P* = 0.049) when compared with AML patients, respectively (**Figure 5**). It was elucidated that EBV reactivation reduced the incidence of relapse in patients with malignant hematological disorders after haplo-HCT, possibly because early viral infection had a direct effect on immune recovery, which in turn reduced the risk of relapse (Klyuchnikov et al., 2010; Janeczko et al., 2016; Gao et al., 2019). Despite these data and hypotheses, the unexplainable increased CIR of AML patients in the EBV+/CMV− subgroup still warranted further validation and investigation.

In conclusion, we elaborated on the features, risk factors, and impacts on the outcomes of EBV and CMV reactivation in acute leukemia patients receiving haplo-HCT with myeloablative conditioning containing ATG. This study had several limitations, including the single-center retrospective nature, diversity of therapeutic interventions for viral reactivations, bias in pre- and posttransplant treatment regimens, and limited sample size, which may affect the reliability of the statistical analyses. Furthermore, the pattern of immunoreconstitution was not analyzed due to insufficient data on the dynamic monitoring of lymphocyte subsets. Because our retrospective study focused on recipients after haplo-HCT with an ATG-contained regimen, the impact of serotherapy should be taken into account in further studies (Lindemans et al., 2015; Storek, 2015; Willemsen et al., 2015; Gergely et al., 2021; Keogh et al., 2021). Hence, our findings need to be validated by large-scale real-world studies and laboratory investigations in the future.

## Data Availability Statement

The raw data supporting the conclusions of this article will be made available by the authors, without undue reservation.

## Ethics Statement

Written informed consent was obtained from the individual(s), and minor(s)’ legal guardian/next of kin, for the publication of any potentially identifiable images or data included in this article.

## Author Contributions

JC and DW: conception and design of the study. YR and JZ: data acquisition, analysis, and interpretation. YR and TS drafted the article and critically revised the manuscript for important intellectual content. DW: gave the final approval of the version to be submitted. All authors contributed to the article and approved the submitted version.

## Conflict of Interest

The authors declare that the research was conducted in the absence of any commercial or financial relationships that could be construed as a potential conflict of interest.

## Publisher’s Note

All claims expressed in this article are solely those of the authors and do not necessarily represent those of their affiliated organizations, or those of the publisher, the editors and the reviewers. Any product that may be evaluated in this article, or claim that may be made by its manufacturer, is not guaranteed or endorsed by the publisher.

## Supplementary Material

Supplementary Figure 1 Transplant outcomes between AML and ALL patients in the whole cohort. **(A)** OS between AML and ALL patients in the whole cohort. **(B)** PFS between AML and ALL patients in the whole cohort. **(C)** TRM between AML and ALL patients in the whole cohort. **(D)** CIR between AML and ALL patients in the whole cohort.
