# Supplementary figures and images for: Features of Epstein–Barr Virus and Cytomegalovirus Reactivation in Acute Leukemia Patients After Haplo-HCT With Myeloablative ATG-Containing Conditioning Regimen
Source: Front Cell Infect Microbiol. 2022 May 16;12:865170. doi: 10.3389/fcimb.2022.865170 (PMC9149257; doi:10.3389/fcimb.2022.865170)

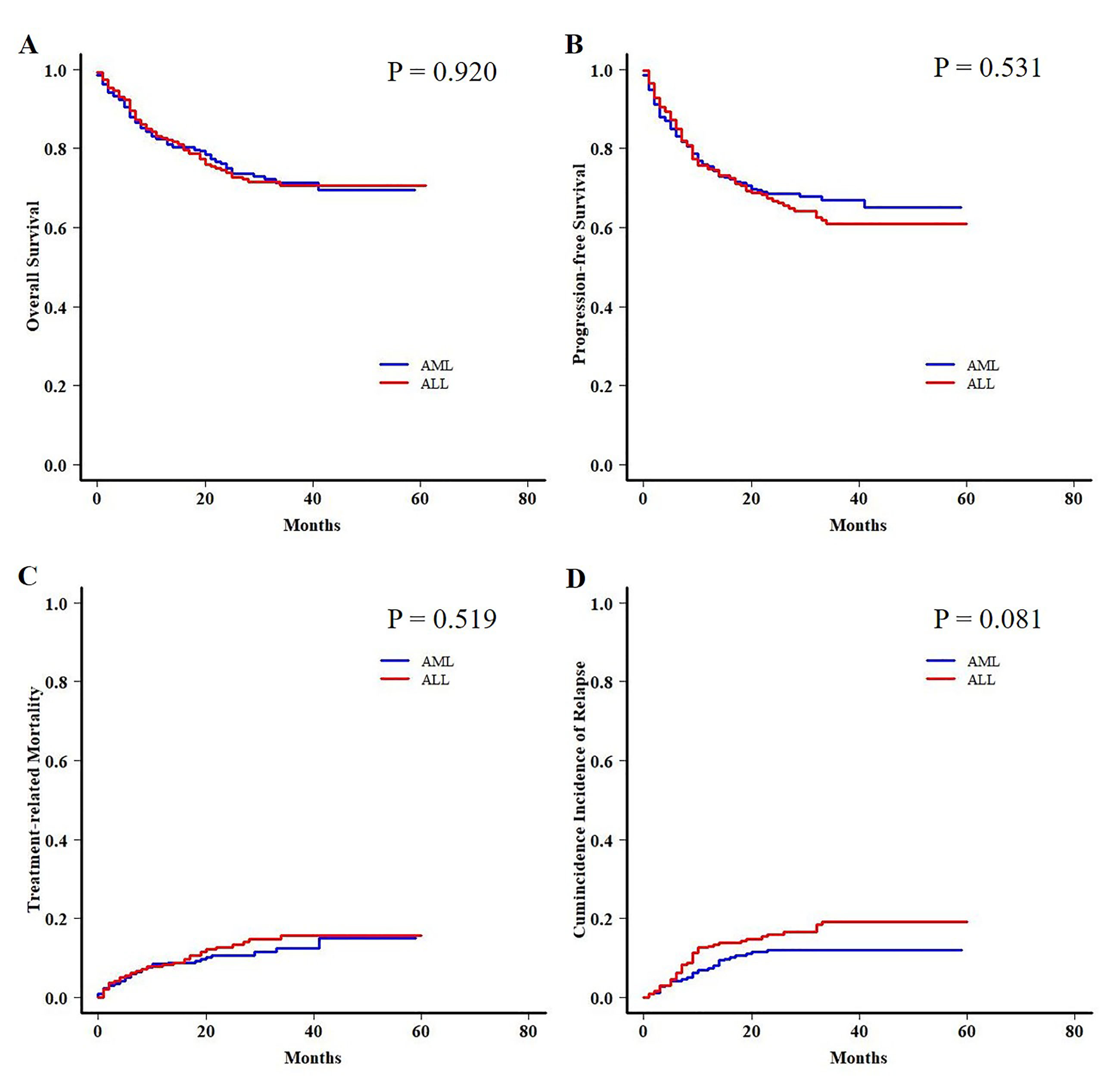

Supplement: Supplementary Figure 1 — Transplant outcomes between AML and ALL patients in the whole cohort. (A) OS between AML and ALL patients in the whole cohort. (B) PFS between AML and ALL patients in the whole cohort. (C) TRM between AML and ALL patients in the whole cohort. (D) CIR between AML and ALL patients in the whole cohort. [file Image_1.tif]
