# Supplementary material for: Features of Epstein–Barr Virus and Cytomegalovirus Reactivation in Acute Leukemia Patients After Haplo-HCT With Myeloablative ATG-Containing Conditioning Regimen
Source: Front Cell Infect Microbiol. 2022 May 16;12:865170. doi: 10.3389/fcimb.2022.865170 (PMC9149257; doi:10.3389/fcimb.2022.865170)
Supplement: Supplementary file 2 [file DataSheet_1.docx]

Supplementary Material

**Table S1. Univariate Cox regression model about association between variables and EBV reactivation in AML patients**

| **Factors** |  | **EBV+**  **cases** | **EBV–**  **cases** | **P** | **HR（95%CI）** |
| --- | --- | --- | --- | --- | --- |
| **Age (years)** | **< 25** | 29 | 81 | 0.560 | 1.146（0.725 – 1.810） |
|  | **≥ 25** | 50 | 171 |  |  |
| **Period** | **Adult** | 64 | 200 | 0.642 | 1.143（0.651 – 2.005） |
|  | **Children** | 15 | 52 |  |  |
| **Sex** | **Female** | 24 | 120 | 0.014 | 1.824（1.129 – 2.946） |
|  | **Male** | 55 | 132 |  |  |
| **Disease status before HCT** | **CR1 or CR2** | 65 | 202 | 0.925 | 0.973（0.546 – 1.734） |
|  | **CR3 or beyond** | 14 | 50 |  |  |
| **Acute GVHD** | **Grade 0-1** | 53 | 196 | 0.057 | 1.602（0.985 – 2.605） |
|  | **Grade 2-4** | 26 | 56 |  |  |
| **Chronic GVHD** | **Absent** | 57 | 166 | 0.392 | 1.484（0.601 – 3.668） |
|  | **Present** | 22 | 86 |  |  |
| **CMV reactivation** | **Positive** | 42 | 43 | <0.001 | 3.751（2.369 – 5.941） |
|  | **Negative** | 37 | 209 |  |  |

EBV: Epstein-Barr virus; CMV: cytomegalovirus; AML: acute myeloid leukemia; HCT: hematopoietic cell transplantation; GVHD: graft-versus-host disease; CR: complete remission; HR: hazard ratio

**Table S2. Univariate Cox regression model about association between variables and CMV reactivation in AML patients**

| **Factors** |  | **CMV+**  **cases** | **CMV–**  **cases** | **P** | **HR（95%CI）** |
| --- | --- | --- | --- | --- | --- |
| **Age (years)** | **< 25** | 27 | 83 | 0.633 | 0.895（0.567 – 1.413） |
|  | **≥ 25** | 58 | 163 |  |  |
| **Period** | **Adult** | 71 | 193 | 0.288 | 1.364（0.769 – 2.421） |
|  | **Children** | 14 | 53 |  |  |
| **Sex** | **Female** | 27 | 117 | 0.024 | 1.694（1.073 – 2.675） |
|  | **Male** | 58 | 129 |  |  |
| **Disease status before HCT** | **CR1 or CR2** | 72 | 195 | 0.534 | 0.829（0.459 – 1.497） |
|  | **CR3 or beyond** | 13 | 51 |  |  |
| **Acute GVHD** | **Grade 0-1** | 55 | 194 | 0.014 | 1.780（1.122 – 2.825） |
|  | **Grade 2-4** | 30 | 52 |  |  |
| **Chronic GVHD** | **Absent** | 58 | 165 | 0.336 | 1.697（0.577 – 4.989） |
|  | **Present** | 27 | 81 |  |  |
| **EBV reactivation** | **Positive** | 42 | 37 | < 0.001 | 3.948（2.531 – 6.156） |
|  | **Negative** | 43 | 209 |  |  |

CMV: cytomegalovirus; EBV: Epstein-Barr virus; AML: acute myeloid leukemia; HCT: hematopoietic cell transplantation; GVHD: graft-versus-host disease; CR: complete remission; HR: hazard ratio

**Table S3. Univariate Cox regression model about association between variables and EBV reactivation in ALL patients**

| **Factors** |  | **EBV+**  **cases** | **EBV–**  **cases** | **P** | **HR（95%CI）** |
| --- | --- | --- | --- | --- | --- |
| **Age (years)** | **< 25** | 45 | 107 | 0.669 | 1.104（0.702 – 1.737） |
|  | **≥ 25** | 32 | 87 |  |  |
| **Period** | **Adult** | 47 | 121 | 0.906 | 0.973（0.615 – 1.538） |
|  | **Children** | 30 | 73 |  |  |
| **Sex** | **Female** | 29 | 73 | 0.990 | 1.003（0.632 – 1.590） |
|  | **Male** | 48 | 121 |  |  |
| **Disease status before HCT** | **CR1 or CR2** | 70 | 176 | 0.998 | 1.001（0.460 – 2.178） |
|  | **CR3 or beyond** | 7 | 18 |  |  |
| **Acute GVHD** | **Grade 0-1** | 60 | 138 | 0.862 | 0.953（0.556 – 1.636） |
|  | **Grade 2-4** | 17 | 56 |  |  |
| **Chronic GVHD** | **Absent** | 54 | 121 | 0.046 | 3.028（1.020 – 8.988） |
|  | **Present** | 23 | 73 |  |  |
| **CMV reactivation** | **Positive** | 36 | 46 | 0.003 | 2.069（1.289 – 3.319） |
|  | **Negative** | 41 | 148 |  |  |

EBV: Epstein-Barr virus; CMV: cytomegalovirus; ALL: acute lymphoblastic leukemia; HCT: hematopoietic cell transplantation; GVHD: graft-versus-host disease; CR: complete remission; HR: hazard ratio

**Table S4. Univariate Cox regression model about association between variables and CMV reactivation in ALL patients**

| **Factors** |  | | **CMV+**  **cases** | **CMV–**  **cases** | **P** | **HR（95%CI）** |
| --- | --- | --- | --- | --- | --- | --- |
| **Age (years)** | **< 25** | | 43 | 109 | 0.413 | 0.834（0.541 – 1.287） |
|  | **≥ 25** | | 39 | 80 |  |  |
| **Period** | | **Adult** | 55 | 113 | 0.249 | 1.312（0.827 – 2.079） |
|  |  | **Children** | 27 | 76 |  |  |
| **Sex** | **Female** | | 32 | 70 | 0.648 | 0.902（0.597 – 1.405） |
|  | **Male** | | 50 | 119 |  |  |
| **Disease status before HCT** | **CR1 or CR2** | | 72 | 174 | 0.206 | 1.534（0.791 – 2.974） |
|  | **CR3 or beyond** | | 10 | 15 |  |  |
| **Acute GVHD** | **Grade 0-1** | | 52 | 146 | 0.393 | 1.246（0.752 – 2.064） |
|  | **Grade 2-4** | | 30 | 43 |  |  |
| **Chronic GVHD** | **Absent** | | 50 | 125 | 0.085 | 2.939（0.863 – 10.007） |
|  | **Present** | | 32 | 64 |  |  |
| **EBV reactivation** | **Positive** | | 36 | 41 | 0.007 | 1.979（1.205 – 3.250） |
|  | **Negative** | | 46 | 148 |  |  |

CMV: cytomegalovirus; EBV: Epstein-Barr virus ;ALL: acute lymphoblastic leukemia; HCT: hematopoietic cell transplantation; GVHD: graft-versus-host disease; CR: complete remission; HR: hazard ratio

**Table S5. Comparisons of transplant outcomes between AML and ALL patients**

|  |  | 2-year OS | 2-year PFS | 2-year TRM | 2-year CIR |
| --- | --- | --- | --- | --- | --- |
| Whole cohort | AML | 75.1% ± 2.5% | 68.5% ± 2.6% | 10.6% ± 0.0% | 12.1% ± 0.0% |
|  | ALL | 73.9% ± 2.8% | 66.7% ± 3.0% | 12.6% ± 0.0% | 15.9% ± 0.1% |
|  | P | 0.920 | 0.531 | 0.519 | 0.081 |
| EBV+/CMV- subgroup | AML | 60.3% ± 8.8% | 57.5% ± 8.4% | 6.9% ± 0.2% | 32.4% ± 0.8% |
|  | ALL | 82.0% ± 6.2% | 74.5% ± 7.0% | 13.6% ± 0.3% | 8.2% ± 0.2% |
|  | P | 0.016 | 0.036 | 0.666 | 0.010 |

AML: acute myeloid leukemia; ALL: acute lymphoblastic leukemia; EBV: Epstein-Barr virus; CMV: cytomegalovirus; OS: overall survival; PFS: progression-free survival; TRM: treatment-related mortality; CIR: cumulative incidence of relapse;

**Table S6. Univariate Fine and Gray model for CIR in EBV+/CMV- subgroup**

| **Factors** |  | **Cases** | **P** | **HR（95%CI）** |
| --- | --- | --- | --- | --- |
| **Age (years)** | **< 25** | 37 | 0.154 | 2.411(0.720 – 8.077) |
|  | **≥ 25** | 41 |  |  |
| **Sex** | **Female** | 28 | 0.921 | 1.059(0.338 – 3.320) |
|  | **Male** | 50 |  |  |
| **Period** | **Adult** | 51 | 0.106 | 2.658(0.831 – 8.503) |
|  | **Children** | 27 |  |  |
| **Disease** | **AML** | 37 | 0.044 | 3.135(1.032 – 9.528) |
|  | **ALL** | 41 |  |  |
| **Disease status before HCT** | **CR1 or CR2** | 67 | 0.167 | 2.495(0.682 – 9.123) |
|  | **CR3 or beyond** | 11 |  |  |
| **Acute GVHD** | **Grade 2-4** | 19 | 0.862 | 1.122(0.307 – 4.108) |
|  | **Grade 0-1** | 59 |  |  |
| **Chronic GVHD** | **Absent** | 56 | 0.206 | 0.381(0.085 – 1.701) |
|  | **Present** | 22 |  |  |

CIR: cumulative incidence of relapse; EBV: Epstein-Barr virus; CMV: cytomegalovirus; HCT: hematopoietic cell transplantation; GVHD: graft-versus-host disease; AML: acute myeloid leukemia; ALL: acute lymphoblastic leukemia; CR: complete remission; HR: hazard ratio
